# Adolescent Obesity and Charlson Comorbidity Index in Young Adults

**DOI:** 10.3390/jcm14030873

**Published:** 2025-01-28

**Authors:** Yulia Treister-Goltzman, Idan Menashe, Dan Nemet

**Affiliations:** 1 Department of Family Medicine and Siaal Research Center for Family Practice and Primary Care, The Haim Doron Division of Community Health, Faculty of Health Sciences, Ben-Gurion University of the Negev, P.O. Box 653, Beer-Sheva 84105, Israel; 2 Clalit Health Services, Southern District, P.O. Box 16250, Beer-Sheva 84161, Israel; 3 Department of Epidemiology, Biostatistics, and Community Health Sciences, Faculty of Health Sciences, Ben-Gurion University of the Negev, Beer-Sheva 84105, Israel; idanmen@bgu.ac.il; 4 Child Health and Sports Center, Meir Medical Center, Kfar-Saba 4428164, Israel; 5 School of Medicine, Tel Aviv University, Tel Aviv 6139001, Israel; dan.nemet@clalit.org.il

**Keywords:** adolescent obesity, morbidity, mortality, Charlson comorbidity index

## Abstract

**Background:** There is insufficient evidence regarding the independent risk of childhood/adolescent obesity for morbidity and mortality in adulthood. The objective of the present study was to evaluate the association of weight categories during adolescence with high-risk diseases determined by the Charlson Comorbidity Index in young adulthood. We also analyzed the association of weight categories with cumulative mortality at the age of 30. **Methods:** A retrospective cohort study, based on the central computerized database of a major health service organization, was conducted. The study population consisted of 80,853 adolescents. The study period was from 1 January 2007 to 31 December 2022 and was divided into the exposure period from 1 January 2007 to 31 December 2011 (ages 17–19) and the follow-up period from 1 January 2007 to 31 December 2022 (from the date of the defining BMI measurement up to the age of 30 years). **Results:** The five diseases with the highest cumulative incidence were chronic pulmonary disease (8.2%), mild liver disease (3.7%), cerebrovascular disease (2.8%), diabetes without end-organ damage (2.0%), and peptic disease (1.6%). When adjusted for socio-demographic variables and adult BMI, the relative risks with 95% confidence intervals for the increase in the Charlson Comorbidity Index were 1.11 (1.05–1.17), 1.17 (1.11–1.24), and 1.22 (1.09–1.35) for the “overweight”, “obesity”, and “class 2 obesity” categories, respectively, while the mortality for these categories were 1.60 (1.11–2.27), 1.71 (1.12–2.57), and 3.18 (1.48–6.35), respectively. **Conclusions:** Adolescent obesity is an independent risk factor for high-risk diseases and mortality in young adulthood. Interventions aimed at reducing the rate of adolescent overweight and obesity should be implemented as early as possible.

## 1. Introduction

Adolescent obesity has been shown to be associated with multiple diseases in adulthood, as young as 30 years of age and below. The most studied consequences of adolescent obesity in adulthood are diabetes mellitus type 2 [1,2], hypertension [3,4], metabolic syndrome [5], and malignancy [6,7]. Less rigorous evidence exists for an association of adolescent obesity with renal failure, diabetes type 1 [8], and cardiovascular end-organ damage in adulthood, such as ischemic heart disease, myocardial infarction, stroke, heart failure, and mortality [9,10,11,12,13,14,15,16]. A lack of convincing evidence for the latter stems from the need for longer prospective studies for the identification of these conditions, which usually occur later in life. Several recent systematic reviews and meta-analyses on the topic concluded that, currently, there is insufficient evidence for an independent risk of childhood BMI for the established cardiovascular diseases and mortality in adulthood [14,15,17], with the increased risk usually attributed to adult obesity. These reviews emphasized the need for more robust epidemiological data on the long-term risk of adolescent obesity in adulthood. Additionally, the association of adolescent obesity with concurrent and future Metabolic-associated Fatty Liver Disease, which, in turn, is independently associated with cardiovascular risks, is well-researched [18]. There also are reports on the less well-acknowledged association of adolescent obesity with peptic diseases [19].

A recent study analyzed changes in the list of the top causes of death [20], and all of the leading causes, including heart disease, cancer, chronic respiratory disease, cerebrovascular disease, diabetes mellitus, and chronic renal disease, have been linked to adolescent obesity in the scientific literature, but with different strengths of evidence.

There are reports of an increasing prevalence of adolescent obesity during the COVID-19 pandemic [21] in most countries around the world. The growing body of evidence linking adolescent obesity to multiple serious morbidities in adulthood, comprising the leading causes of death, highlights the importance of further research on the impact of adolescent obesity on long-term morbidity and mortality. Given the difficulties of assessing the direct long-term health consequences of adolescent obesity, including mortality, we decided to use a proxy measure of future mortality risk, i.e., the Charlson Comorbidity Index (CCI), for this purpose.

The main goal of this study was to evaluate the association of weight categories during adolescence with the CCI in young adulthood. An additional goal was to analyze the association of weight categories with cumulative mortality at the age of 30.

## 2. Methods

This was a retrospective cohort study based on the central computerized database of Clalit Health Services (CHS), a major health service organization that insures 52% of the Israeli population. The study population consisted of adolescents born in 1988–1992 who had measurements of weight and height at ages 17–19 years in their medical records. Participants with major chromosomal anomalies and/or intellectual disabilities were excluded from the cohort. Since we assessed the risk for future mortality through the development of high-risk comorbidity up to the age of 30 years, we also excluded from the main cohort adolescents who had CCI-related diseases at the baseline (i.e., to assure that the outcome did not occur before the exposure), participants who died before the age of 30 years, and participants who discontinued insurance in CHS before age 30. Only participants who discontinued insurance in CHS before age 30 were excluded from the cohort for the analysis of the actual mortality at the ages of 30 years and below.

The study period was 1 January 2007 to 31 December 2022 and was divided into the exposure period—1 January 2007 to 31 December 2011 (ages 17–19)—and the follow-up period—1 January 2007 to 31 December 2022 (from the date of the defining BMI measurement up to the age of 30 years). Data that were collected included socio-demographic data (age, sex, ethnic sector, and district of residence), socio-economic status (as defined in the Clalit computerized database by zip code), dates of insurance termination in CHS, height (cm), weight (kg), BMI, the diagnoses that make up the Charlson index, date of death (if death occurred), and major chromosomal anomalies and intellectual disabilities (Table S1).

The main exposure variable was adolescent weight categories defined as percentiles determined by the U.S. Center for Disease Control and Prevention (CDC), which were validated for Israeli adolescents [22,23] (Table S1). The main outcome variable was a CCI score of 19 chronic conditions that impact survival outcome, especially long-term survival [24]. Although the CCI is often used to predict disability, quality of life, health care costs, and hospitalization, it was originally designed to predict long-term mortality [24]. It is the most widely used comorbidity index for the determination of survival rate [24]. It includes chronic conditions strongly associated with obesity, such as diabetes, a range of cardiovascular diseases and their complications, pulmonary and liver diseases, and malignancy. For most of these diseases, a stepwise gradual increase in risk has been demonstrated with increasing obesity severity [1,2,3,4,5,6,7,8,9,10,11,12,13]. Thus, we presumed that the CCI would enable us to conduct a comprehensive assessment of mortality risk, on the one hand, and dose–response analyses across weight categories, on the other. 

Clinimetric properties, a term originally coined by Alvan R. Feinstein, is the ability of a tool to measure clinically important measurements (e.g., inter-rater reliability, concurrent validity, sensitivity, and predictive validity) [25]. Of these, concurrent validity (i.e., a clinimetric property that evaluates the correlation between scales that measure the same concept), sensitivity (i.e., stepwise increases in mortality with stepwise increases in the CCI), and predictive validity (i.e., the ability of a rating scale or index to predict a future outcome and stratify patients into distinctively different prognostic groups) are the most relevant clinimetric CCI properties for our study, and they were validated extensively. Most studies that evaluated the concurrent validity of the CCI found moderate or strong correlations compared to other measures that predict mortality [26,27,28]. Studies that focused on sensitivity showed consistent gradients with increased mortality with an increasing CCI score [29,30,31].

The CCI has been adapted for use with different sources of data, including international classification of diseases 9 and 10 ( ICD-9 and ICD-10) codes [24], and has shown excellent predictive validity in different settings, predicting mortality in a range of 30 days to 10 years [32,33,34].

We used the Deyo at al. [35] adaptation of the CCI to the ICD-9 diagnostic codes, which adhered to strict criteria and showed very good concordance with other versions of the CCI that were adapted to ICD-9 [24] as well as excellent prediction for mortality in direct comparison to actual mortality in epidemiologic studies [36] (Table S1).

### Statistical Analyses

Data cleaning was performed, and outlying BMI values were deleted. We compared the basic socio-demographic characteristics of adolescents with and without BMI measurements for possible selection bias. The final study sample was set following the exclusion of patients with major chromosomal abnormalities and intellectual disabilities. For adolescents with several weight and height measurements at ages 17–19, we chose the earliest ones for further analysis. We used descriptive statistics to characterize the baseline features of the study population.

Two separate analyses were performed. The first one evaluated associations with CCI at age 30 among adolescents from different weight categories. The mean (SD) CCI and a crude cumulative incidence (%) of CCI ≥ 1 and CCI ≥ 3 at age 30 were calculated for adolescents from different weight groups, and the cumulative incidence of the individual diseases comprising the CCI in each weight category was analyzed.

We built a negative binomial regression, which assessed the association of weight categories with the CCI. The “normal” weight category served as the reference group. Negative binomial regression is suited better than Poisson for events in clinical settings in which the variance of events is usually greater than their mean [37]. The Poisson regression models were built to assess the association of weight categories with two CCI cutoffs (≥1 and ≥3), which represent the most researched cutoffs of the index for its association with future mortality. The Poisson regression model [38], which utilizes a sandwich variance estimator, is widely used in prospective studies with binary outcomes. The primary advantage of this approach is that it readily provides covariate-adjusted risk ratios and associated standard errors [38]. Analyses of the association of weight categories with the CCI were carried out separately for males and females.

For the additional goal of our study, i.e., the analysis of RRs (95% CIs) for cumulative all-cause mortality at ages 30 years and below among adolescents from different weight categories, we also used Poisson regression models. We did not use Cox proportional hazard models because the proportionality assumption was violated since the curves of the ‘log minus log’ plots intersected, and the results of the global Schoenfeld residual test were significant.

All multivariable models were adjusted for sex, ethnicity, socio-economic status, district of residency, and adult BMI. Statistical significance was set at *p* < 0.05.

The Ethics Committee of Clalit Health Services approved this study (approval #0102-22-COM2, date: 11 December 2022) and exempted it from the requirement to obtain informed consent.

## 3. Results

**Figure 1:**
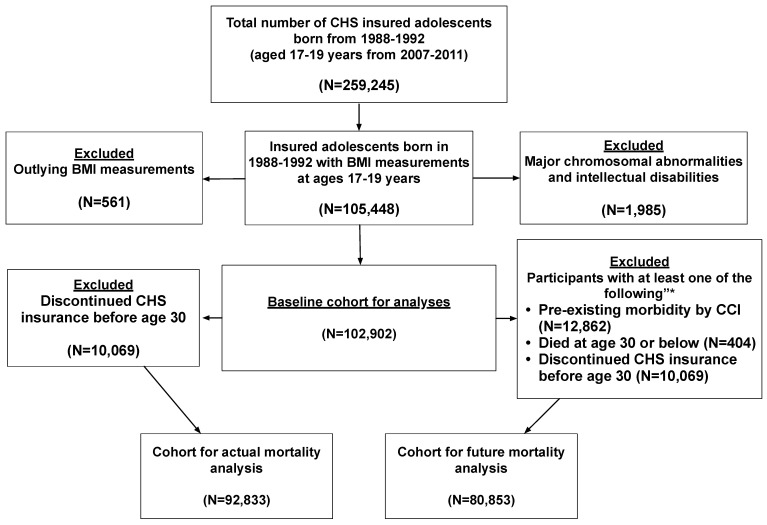
Flowchart of the study cohort selection. * Several options are possible for the same participant.

A flowchart of the study selection process is shown in Figure 1. Of 259,245 adolescents insured by CHS, who reached ages 17–19 in 2007–2011, 105,448 adolescents had recorded weight and height measurements. After excluding individuals with outlying BMI values, we compared the socio-demographic characteristics of the 104,887 remaining adolescents to those of adolescents with no BMI measurements or with outlying BMI measurements (Table S2). There were fewer male adolescents with measurements (47.3% vs. 51.7%) and more adolescents in the lower socio-economic class than those without measurements (36.5% vs. 31.4%).

### 3.1. Association of Adolescent Weight Categories with CCI at Age 30

Table 1 presents the socio-demographic characteristics of the cohort for the evaluation of the associations of the weight categories with the CCI at the age of 30 years. Baseline differences justified the adjustment made to the multivariable models.

Table 2 presents details of the CCI score among adolescents from different weight categories and the association of weight categories with this score. The cumulative prevalence of adolescents who had at least one disease included in the CCI increased consistently from the “underweight” to the “class 3 obesity” category, even more so for adolescents with a CCI of at least three (from 14.4% to 38.7% and from 0.5% to 3.2%). Compared to adolescents in the “normal” weight category, the RR (95% CI) for the increase in the CCI rose to 1.71 in the overweight category (1.63–1.80) and continued to increase up to 2.51 (2.17–2.90) in the “class 3 obesity” category. After adjusting for adult BMI, the aRRs (95% CI) decreased but remained significant in all but the “class 3 obesity” category. When analyzed by gender, the total population risk measurements were comparable, except for significantly higher RRs (95% CI) for the CCI in the “class 3 obesity” category among males 1.37 (1.07–1.76) but not among females 0.94 (0.76–1.14) in the fully adjusted models. A similar increase in aRR was observed with a dichotomic dependent variable of CCI ≥ 1 (Table S3). In models with the dependent variable of CCI *≥* 3, RR (95% CI) gradually increased to 4.52 (2.75–6.94) in the “class 3 obesity” category when adjusted for socio-demographic variables only. The graded association was less evident when adjusted for adult BMI as well. Compared to the “normal” weight category, the risk was increased in the “overweight” and “class 2 obesity” categories (RRs (95% CI) = 1.25 (1.01–1.55) and 1.88 (1.31–2.64), respectively) (Table S3).

**Figure 2:**
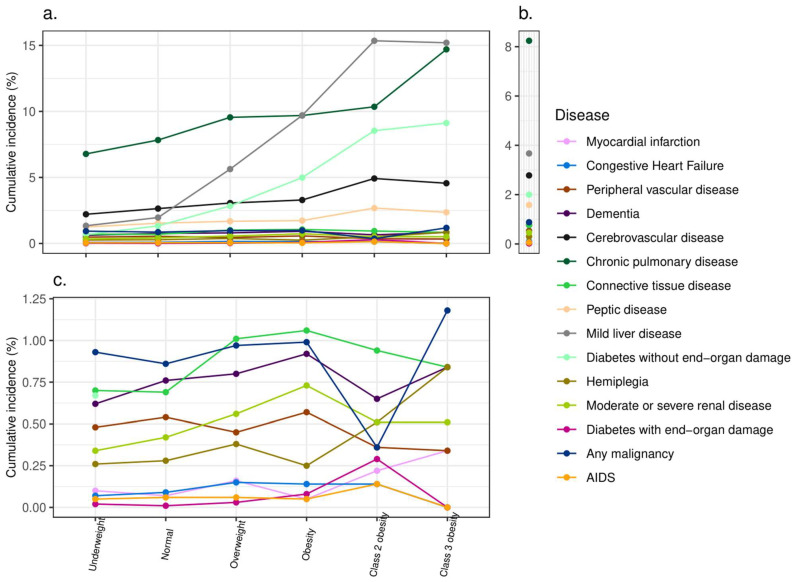
Cumulative incidence of individual diseases composing the Charlson Comorbidity Index in early adulthood among adolescents from different weight categories. (**a**) Cumulative incidence (%) per weight category. (**b**) Overall cumulative incidence (%). (**c**) Conditions with low cumulative incidence (less than 1%). Underweight—BMI < 5th percentile, normal weight—BMI 5th–84.9th percentile, overweight—BMI 85th–94.9th percentile, obesity—BMI ≥ 95th percentile, not including class 2 and class 3 obesity, class 2 obesity—BMI ≥ 120% to <140% of the 95th percentile or BMI ≥ 35 to <40 kg/m^2^, and class 3 obesity—BMI ≥ 140% of the 95th percentile or BMI ≥ 40 kg/m^2^.

Figure 2 and Table S4 present the cumulative incidence, among weight categories, of the individual diseases that compose the CCI. The five diseases with the highest cumulative incidence were chronic pulmonary disease (6663 (8.2%)), mild liver disease (2294 (3.7%)), cerebrovascular disease (2251 (2.8%)), diabetes without end-organ damage (1619 (2.0%)), and peptic disease (1276 (1.6%)). No participant with metastatic solid tumor and moderate to severe liver disease was in the cohort at age 30 (since one of the inclusion criteria was being alive at age 30, which is less probable with these diagnoses). Diseases with a low prevalence rate included diabetes with complications (20 (0.02%)), AIDS (47 (0.06%)), and myocardial infarction (66 (0.08%)). Most of the diseases were more prevalent in one or more of the excessive weight categories (“overweight”, “obesity”, “class 2 obesity”, and “class 3 obesity”) than in the normal weight category, except for peripheral vascular disease, for which prevalence was slightly lower in most of the excess weight categories. For the five conditions with the highest incidence, a clear dose-dependent increase in prevalence with an increase in the weight category was observed.

### 3.2. Association of Weight Categories with All-Cause Mortality

Of 92,833 adolescents in the cohort for all-cause mortality estimation, 404 (0.4%) died at ages 30 and below (Table 3). The crude cumulative incidence of all-cause mortality increased from the “normal” to “class 2 obesity” category from 0.4% to 0.8%. Adult BMI also increased in the adolescent weight categories. Compared to the “normal” weight category, the RR (95% CI) for all-cause mortality was increased only in the “class 2 obesity” category in the unadjusted and adjusted for sex, ethnicity, socio-economic level, and district of residency models. When further adjusted for adult BMI, the risk increased in the “overweight” category (aRR (95% CI) = 1.60 (1.11–2.27)), reaching an aRR (95% CI) of 3.18 (1.48–6.35) in the “class 2 obesity” category.

## 4. Discussion

The uniqueness and novelty of the present study is that it assessed potential associations of adolescent weight categories with a spectrum of high-risk diseases that pose a risk for both short- and long-term mortality at the age of 30 years. The association of adolescent obesity with diseases such as hypertension and diabetes mellitus type 2 is already apparent in adolescence [12]. Although several studies have linked adolescent obesity with high-risk morbidity in adulthood, most of them were criticized for the lack of adjustment for adult BMI [1,7,11,16]. Since most adolescents with overweight and obesity continue with these conditions in adulthood [39], the lack of adjustment for adult BMI casts doubt on the causal nature of the associations found in the studies. In the present study, the increase in the adolescent weight category was already associated with an increase in the CCI at age 30. This finding did not change after adjustment for socio-demographic variables and adult BMI. The composition of the CCI at age 30 is interesting. The two diseases with the highest cumulative incidence in the total study cohort and among adolescents with obesity were chronic pulmonary disease and mild liver disease. This overall high incidence is not surprising and correlates with the known reported high prevalence of these diseases in young adults [40,41,42]. An increase in their prevalence and even a strong graded relationship between BMI and chronic obstructive pulmonary disease, as well as asthma, have been reported in several studies [43,44]. This may be a consequence of altered lung and chest wall mechanics and an increased production of inflammatory cytokines and immune cells that play a role in multiple pulmonary diseases [45]. Although some studies propose a protective role of obesity on morbidity from chronic lung disease, more recent evidence shows that this phenomenon does not pertain to the extremes of obesity (BMI > 40) [46]. The liver impairment most closely associated with obesity in adulthood is Metabolic-associated Fatty Liver Disease [41]. Studies have shown its high prevalence and positive association with BMI among adolescents too [47,48]. Rare liver diseases such as autoimmune and toxic injury were also shown to be associated with overweight and obesity in a recent study [42]. Although the increase in crude prevalence was observed for pulmonary and mild liver diseases, a relatively high prevalence of pulmonary diseases was observed in lower weight categories as well, whereas, for mild liver diseases, this prevalence rose steeply in the overweight and obesity categories. The association of weight categories with cerebrovascular diseases at ages 30 and below has been demonstrated for all strokes, including ischemic stroke, even without other known risk factors [11,49], and a large body of evidence exists regarding their association with diabetes mellitus [1,2]. Epidemiological evidence relating obesity to peptic ulcer disease has been mixed, from studies with a negative association [50] to studies with no association [51] and a positive association [19,52]. In our study, there was a twofold increase in the incidence of peptic disease from the “underweight” to “class 2” and “class 3” obesity categories. Among diseases with a cumulative incidence of less than 1%, the increase in incidence in weight categories was less consistent, although increased incidence was observed in one or more excessive weight categories for all diseases, with the exception of peripheral vascular disease. Indeed, these diseases share common key pathophysiological mechanisms, particularly abnormalities in adipokines, the aberrant fatty acid uptake phenotype, inflammation and hyperinsulinemia, and even the genetic and molecular factors in the context of obesity [53,54]. As for peripheral vascular disease, a recent in-depth review suggested a potential protective role of higher BMI, the so-called “obesity paradox”, which presumably stems from body fat distribution rather than adiposity, with lower body white adipose tissue having more anti-inflammatory properties [55]. These common trends in individual components of the CCI, represented by weightcategories, resulted in a dose-dependent increase in CCI ≥ 1 and ≥3, which are the two most researched cutoffs that are closely associated with short and long-term mortality [24], as well as with an increase in RR with the CCI as a continuous numeric dependent variable.

An additional important finding was the increased risk for actual all-cause mortality at a young age, which started from the “overweight” category and consistently increased to the “class 2 obesity” category. A few studies that explored the association of adolescent BMI with mortality focused predominantly on adults who were middle-aged and older [56,57]. A large-scale study from Israel, which accounted for early adulthood all-cause mortality [58], demonstrated an elevated risk, primarily from cardiovascular causes, with an increase in the weight category but did not adjust for adult BMI, an important confounder. Another study performed by our research team found an increased risk for the composite endpoint of ischemic stroke, myocardial infarction, and heart failure, the major diseases associated with mortality at ages 30 and younger in overweight adolescents [59]. The lack of the association of “class 3 obesity” with mortality and the CCI in the fully adjusted models might stem from the relatively small number of outcome cases in this category. Alternatively, this extreme degree of obesity might be paradoxically associated with some protective effect.

The findings of our study underline the strong need to begin the implementation of interventions designed to reduce overweight and obesity rates in childhood and adolescence. Government-level policymakers and local healthcare and educational authorities should promote the best evidence-based interventions. Increasing hours of physical activity and, to a lesser degree, nutritional interventions are the most successful interventions on a school level according to a comprehensive recent meta-analysis [60]. Interventions adapted to the relevant cultural context, in high-risk pediatric minority populations with personalized physical activity, and closer engagement with families are the most effective programs not only for reducing BMI but also for reversing secondary cardiometabolic obesity risks, including those related to cardiorespiratory capacity, blood pressure, and glucose/insulin levels [61].

### 4.1. Limitations and Strengths

Using BMI measurements from medical records as the definition of exposure may have led to a selection bias. However, we adjusted for these socio-demographic characteristics to minimize that potential bias. Since the recommendation to measure height and weight for the BMI of all adolescents aged 14–19 years has been an integral part of the Israel National program for quality indicators in the community since 2007, the missing BMI measurements could be considered as ‘missing at random.’ Data on the study comorbidities were based on recorded diagnoses. We believe that the use of the validated and reliable version of the CCI adapted for ICD-9 diagnostic codes mitigated the limitations of this approach. Another important limitation is the lack of adjustment for laboratory analyses, such as lipid profile, particularly LDL, which has been shown to be associated with multiple cardiovascular diseases and cancer [62,63]. This study was based on recorded diagnoses; so, in a large nationwide database, it was not possible to extract information on some important variables, such as family history. The COVID pandemic in Israel, at the end of the follow-up period (2020–2022), certainly impacted BMI and mortality rates, but no adjustment was made for this factor in the present study.

The main strength of this study is that it was based on a nationwide, representative, large, and reliable database, with a large number of observations. The measurements of weight and height and the recording of diagnosis were performed by healthcare professionals and were not self-reported. By analyzing different excessive weight categories separately, it was possible to characterize associations with different degrees of obesity. In the present study, we adjusted for adult BMI to overcome the main weakness of previous studies. A historically prospective design of this study, where the data on exposure were documented before the outcome, enabled us to determine the direction of the association.

### 4.2. Conclusions

Even after adjustment for adult BMI, the CCI score already had a clear and dose-dependent association with excess weight in early adulthood (ages 30 and below). The same was true regarding actual early all-cause mortality, although no increase in risk was observed for the “class 3 obesity” category. These findings signify that adolescent obesity is an independent risk factor for mortality from adult BMI and that interventions aimed at reducing the rate of adolescent overweight and obesity should be implemented as early as possible.

## Figures and Tables

**Table 1:** Baseline characteristics of the study cohort.

	Weight Category							
	Underweight (N = 4172)	Normal (N = 57,867)	Overweight (N = 8883)	Obesity (N = 7958)	Class 2 Obesity (N = 1381)	Class 3 Obesity (N = 592)	*p*	Total Population (N = 80,853)
Age of BMI measurement, years								
Mean (SD)	18.0 (0.7)	18.2 (0.8)	18.2 (0.9)	18.0 (0.7)	18.2 (0.9)	18.2 (0.9)	<0.001	18.1 (0.8)
Median (IQR)	17.9 (1.1)	18.0 (1.4)	18.0 (1.5)	17.8 (1.1)	18.0 (1.4)	18.1 (1.5)		17.9 (1.3)
Sex (male)								
N (%)	2195 (52.6)	26,176 (45.2)	4097 (46.1)	4180 (52.5)	672 (48.7)	296 (50.0)	<0.001	37,616 (46.5)
Ethnicity N (%)								
Jewish	2635 (63.2)	25,513 (44.1)	3757 (42.3)	3865 (48.6)	708 (51.3)	330 (55.7)	<0.001	36,808 (45.5)
Arab	1537 (36.8)	32,354 (55.9)	5126 (57.7)	4093 (51.4)	673 (48.7)	262 (44.3)		44,045 (54.5)
BMI, kg/m^2^								
Mean (SD)	16.8 (1.0)	21.3 (2.0)	26.5 (1.3)	30.5 (2.1)	36.9 (1.4)	44.6 (4.6)	<0.001	23.0 (4.6)
Median (IQR)	16.9 (1.2)	21.2 (3.2)	26.4 (1.6)	30.3 (3.3)	36.6 (2.3)	43.1 (4.8)		22.0 (45.0)
BMI percentile								
Mean (SD)	2.4 (1.4)	43.5 (23.5)	89.7 (3.0)	97.8 (1.5)	99.3 (1.2)	99.3 (1.3)	<0.001	53.2 (31.5)
Median (IQR)	2.5 (2.3)	44.6 (38.8)	90.0 (5.1)	98.0 (2.6)	99.9 (0.1)	100 (0.0)		51.2 (57.9)
Socio-economic level								
N (%):	438 (10.5)	5229 (9.0)	674 (7.6)	622 (7.8)	87 (6.3)	42 (7.1)	<0.001	7092 (8.8)
High	2250 (53.9)	25,659 (44.3)	3888 (43.8)	3805 (47.8)	678 (49.1)	308 (52.0)		36,588 (45.3)
Middle	1160 (27.8)	22,032 (38.1)	3509 (39.5)	2913 (36.6)	511 (37.0)	200 (33.8)		30,325 (37.5)
Low	324 (7.8)	4947 (8.5)	812 (9.1)	618 (7.8)	105 (7.6)	42 (7.1)		6847 (8.5)
missing								
District of residency								
N (%):	843 (20.2)	7571 (13.1)	1002 (11.3)	1112 (13.8)	189 (13.7)	88 (14.9)	<0.001	10,805 (13.4)
Central	609 (14.6)	10,554 (18.2)	1574 (17.7)	1241 (15.6)	215 (15.6)	71 (12.0)		14,264 (17.6)
Northern	756 (18.1)	11,942 (20.6)	1834 (20.6)	1647 (20.7)	264 (19.1)	119 (20.1)		16,562 (20.5)
Haifa	527 (12.6)	8281 (14.3)	1321 (14.9)	1205 (15.1)	223 (16.1)	87 (14.7)		11,644 (14.4)
Sharon-Shomron	371 (8.9)	3889 (6.7)	588 (6.6)	563 (7.1)	77 (5.6)	58 (9.8)		5546 (6.9)
Dan-PT	322 (7.7)	6913 (11.9)	1266 (14.3)	1052 (13.2)	227 (16.4)	83 (14.0)		9863 (12.2)
Jerusalem Southern	744 (17.8)	8698 (15.0)	1298 (14.6)	1138 (14.3)	186 (13.5)	86 (14.5)		12,150 (15.0)
Missing	0 (0.0)	19 (0.0)	0 (0.0)	0 (0.0)	0 (0.0)	0 (0.0)		19 (0.0)

Underweight—BMI < 5th percentile, normal weight—BMI 5th–84.9th percentile, overweight—BMI 85th–94.9th percentile, obesity—BMI ≥ 95th percentile, not including class 2 and class 3 obesity, class 2 obesity—BMI ≥ 120% to <140% of the 95th percentile or BMI ≥ 35 to <40 kg/m^2^, and class 3 obesity—BMI ≥ 140% of the 95th percentile or BMI ≥ 40 kg/m^2^. SD—standard deviation and IQR—interquartile range.

**Table 2:** Risk estimates of the association between the adolescent weight category and the Charlson Comorbidity Index score at age 30.

	Weight Category						Total Population (N = 80,853)
	Underweight (N = 4172)	Normal (N = 57,867)	Overweight (N = 8883)	Obesity (N = 7958)	Class 2 Obesity (N = 1381)	Class 3 Obesity (N = 592)	
Both sexes							
Charlson score ≥ 1							
N (%)	600 (14.38)	9762 (16.87)	2042 (22.99)	2188 (27.49)	469 (33.96)	229 (38.68)	15,281 (18.90)
Charlson score ≥ 3							
N (%)	21 (0.50)	430 (0.74)	111 (1.25)	122 (1.53)	44 (3.19)	19 (3.21)	747 (0.92)
Charlson score ^a^							
Mean (SD)	1.22 (0.60)	1.26 (0.63)	1.28 (0.65)	1.33 (0.67)	1.42 (0.88)	1.38 (0.73)	1.28 (0.65)
Median (IQR)	1 (0)	1 (0)	1 (0)	1 (0)	1 (1)	1 (1)	1 (0)
Association of weight category in adolescence with Charlson score at age 30							
RR (95% CI) p-value	0.83 (0.76–0.90) <0.001	Reference	1.39 (1.32–1.46) <0.001	1.71 (1.63–1.80) <0.001	2.26 (2.05–2.49) <0.001	2.51 (2.17–2.90) <0.001	
aRR ^b^ (95% CI) p-value	0.85 (0.78–0.93) <0.001		1.39 (1.32–1.46) <0.001	1.76 (1.68–1.84) <0.001	2.30 (2.08–2.53) <0.001	2.59 (2.24–2.98) <0.001	
aRR ^c^ (95% CI) p-value	1.00 (0.92–1.09) 0.940		1.11 (1.05–1.17) <0.001	1.17 (1.11–1.24) <0.001	1.22 (1.09–1.35) <0.001	1.14 (0.98–1.33) 0.090	
b. Males							
Population	2195	26,176	4097	4180	672	296	37,616
Charlson score ≥ 1							
N (%)	258 (11.75)	3510 (13.41)	743 (18.14)	955 (22.85)	201 (29.91)	113 (38.18)	5780 (15.37)
Charlson score ≥ 3							
N (%)	12 (0.55)	175 (0.67)	42 (1.03)	57 (1.36)	19 (2.83)	10 (3.38)	315 (0.84)
Association of weight category in adolescence with Charlson score at age 30							
RR (95% CI) p-value	0.86 (0.76–0.98) 0.021	Reference	1.36 (1.25–1.48) <0.001	1.74 (1.61–1.88) <0.001	2.35 (1.99–2.77) <0.001	3.13 (2.50–3.93) <0.001	
aRR ^b^ (95% CI) p-value	0.87 (0.76–0.98) 0.029		1.36 (1.25–1.47) <0.001	1.74 (1.61–1.88) <0.001	2.37 (2.01–2.79) <0.001	3.14 (2.50–3.94) <0.001	
aRR ^c^ (95% CI) p-value	1.01 (0.88–1.15) 0.894		1.11 (1.02–1.21) 0.014	1.19 (1.09–1.30) <0.001	1.26 (1.05–1.51) 0.010	1.37 (1.07–1.76) 0.011	
c. Females							
Population	1977	31,691	4786	3778	709	296	43,237
Charlson score ≥ 1							
N (%)	342 (17.30)	6243 (19.70)	1299 (27.14)	1233 (32.63)	268 (37.80)	116 (39.19)	9501 (21.97)
Charlson score ≥ 3							
N (%)	9 (0.46)	255 (0.80)	69 (1.44)	65 (1.72)	25 (3.53)	9 (3.04)	432 (1.00)
Association of weight category in adolescence with Charlson score at age 30							
RR (95% CI) p-value	0.84 (0.75–0.93) 0.001	Reference	1.41 (1.33–1.50) <0.001	1.77 (1.66–1.88) <0.001	2.24 (1.98–2.53) <0.001	2.13 (1.76–2.57) <0.001	
aRR ^b^ (95% CI) p-value	0.84 (0.75–0.94) 0.002		1.41 (1.33–1.50) <0.001	1.77 (1.66–1.88) <0.001	2.24 (1.99–2.53) <0.001	2.14 (1.77–2.58) <0.001	
aRR ^c^ (95% CI) p-value	1.00 (0.90–1.12) 0.946		1.10 (1.03–1.17) 0.002	1.15 (1.07–1.23) <0.001	1.17 (1.03–1.34) 0.018	0.94 (0.76–1.14) 0.513	

^a^ Among those with a Charlson score ≥ 1. Underweight—BMI < 5th percentile, normal weight—BMI 5th–84.9th percentile, overweight—BMI 85th–94.9th percentile, obesity—BMI ≥ 95th percentile, not including class 2 and class 3 obesity, class 2 obesity—BMI ≥ 120% to <140% of the 95th percentile or BMI ≥ 35 to <40 kg/m^2^, class 3 obesity—BMI ≥ 140% of the 95th percentile or BMI ≥ 40 kg/m^2^, SD—standard deviation, IQR—interquartile range, 95% CI—95% of the confidence interval, RR—relative risk, and aRR—adjusted relative risk. ^b^ Adjusted to sex, ethnicity, socio-economic level, and district of residency. ^c^ Adjusted to sex, ethnicity, socio-economic level, district of residency, and adult BMI.

**Table 3:** Risk estimates of the association between the adolescent weight category and all-cause mortality in young adulthood.

	Weight Category						
	Underweight	Normal	Overweight	Obesity	Class 2 Obesity	Class 3 Obesity	Total
Adolescents in the category (N)	4826	65,816	10,324	9445	1638	739	92,833
Cases of death, N (%)	27 (0.6)	266 (0.4)	47 (0.4)	50 (0.5)	13 (0.8)	1 (0.1)	404 (0.4)
Age at death, years, Mean (SD)	25.0 (3.2)	24.4 (3.5)	25.7 (3.3)	24.1 (3.2)	24.8 (3.7)	28.3 (-)	24.6 (3.4)
Adult BMI, kg/m^2^, Mean (SD)	20.6 (3.8)	24.8 (4.6)	30.1 (5.0)	34.1 (5.8)	40.1 (6.0)	44.3 (-)	26.6 (6.3)
RR (95% CI) *p*-value	1.38 (0.91–2.02) 0.107	Reference	1.13 (0.82–1.52) 0.452	1.31 (0.96–1.75) 0.080	1.91 (1.04–3.20) 0.023	0.33 (1.02–1.48) 0.275	
aRR ^a^ (95% CI) *p*-value	1.30 (0.86–1.90) 0.190		1.11 (0.81–1.50) 0.502	1.22 (0.89–1.64) 0.191	1.79 (0.98–3.00) 0.040	0.32 (0.02–1.43) 0.259	
aRR ^b^ (95% CI) *p*-value	1.15 (0.69–1.83) 0.564		1.60 (1.11–2.27) 0.010	1.71 (1.12–2.57) 0.011	3.18 (1.48–6.35) 0.002	0.75 (0.04–3.69) 0.785	

Underweight—BMI < 5th percentile, normal weight—BMI 5th–84.9th percentile, overweight—BMI 85th–94.9th percentile, obesity—BMI ≥ 95th percentile, not including class 2 and class 3 obesity, class 2 obesity—BMI ≥ 120% to <140% of the 95th percentile or BMI ≥ 35 to <40 kg/m^2^, class 3 obesity—BMI ≥ 140% of the 95th percentile or BMI ≥ 40 kg/m^2^, and SD—standard deviation. ^a^ Adjusted to sex, ethnicity, socio-economic level, and district of residency. ^b^ Adjusted to sex, ethnicity, socio-economic level, district of residency, and adult BMI.

## Data Availability

The data that support the findings of this study are available from Clalit Health Services. Restrictions apply to the availability of these data, which were used under license for this study. The data are available only with the permission of Clalit Health Services.

## Supplementary Materials

The following supporting information can be downloaded at: https://www.mdpi.com/article/10.3390/jcm14030873/s1, Table S1: Definition of variables. Table S2: Comparison of adolescents with and without BMI measurements. Table S3: Risk estimates of the association between the adolescent weight category and dichotomic divisions of the Charlson Comorbidity Index at age 30. Table S4: Cumulative incidence of individual diseases of the Charlson Comorbidity Index at age 30, by weight category.
