# Supplementary material for: Adolescent Obesity and Charlson Comorbidity Index in Young Adults
Source: J Clin Med. 2025 Jan 28;14(3):873. doi: 10.3390/jcm14030873 (PMC11818213; doi:10.3390/jcm14030873)
Supplement: Supplementary file 1 [file jcm-14-00873-s001.zip › jcm-3438884-supplementary.pdf]

Table S1. Definition of variables.

<table>
<tr><td colspan="3">a. Main exposure variable: adolescent weight categories</td></tr>
<tr><td>Category</td><td colspan="2">Definition</td></tr>
<tr><td>Underweight</td><td colspan="2">BMI<5th percentile</td></tr>
<tr><td>Normal weight</td><td colspan="2">BMI 5th-84.9th percentile</td></tr>
<tr><td>Overweight</td><td colspan="2">BMI 85th-94.9th percentile</td></tr>
<tr><td>Obesity</td><td colspan="2">BMI≥95th percentile, not including Class 2 and Class 3 obesity</td></tr>
<tr><td>Class 2 obesity</td><td colspan="2">BMI ≥120% to <140% of the 95th percentile or BMI ≥35 to <40 kg/m^2</td></tr>
<tr><td>Class 3 obesity</td><td colspan="2">BMI ≥140% of the 95th percentile or BMI ≥40 kg/m^2.</td></tr>
<tr><td colspan="3">b. Scoring the Charlson Comorbidity Index from ICD-9 codes*</td></tr>
<tr><td>Conditions</td><td>Weights</td><td>ICD-9 codes</td></tr>
<tr><td>Myocardial infarction</td><td>1</td><td>410-410.9 and 412</td></tr>
<tr><td>Congestive heart failure</td><td>1</td><td>428-428.9</td></tr>
<tr><td>Peripheral vascular disease</td><td>1</td><td>443.9, 441,441.9, 785.4, and V43.4, procedure 38.48</td></tr>
<tr><td>Dementia</td><td>1</td><td>290-290.9</td></tr>
<tr><td>Cerebrovascular disease</td><td>1</td><td>430-438</td></tr>
<tr><td>Chronic pulmonary disease</td><td>1</td><td>490-496, 500-505, and 506.4</td></tr>
<tr><td>Connective tissue disease</td><td>1</td><td>710.0, 710.1, 710.4, 714.0-714.2, 714.81, and 725</td></tr>
<tr><td>Peptic disease</td><td>1</td><td>531-534.9</td></tr>
<tr><td>Mild liver disease</td><td>1</td><td>571.2, 571.4-571.49, 572.5, 571.6, 571.8, 571.9, and 573</td></tr>
<tr><td>Diabetes without end-organ damage</td><td>1</td><td>250-250.3 and 250.7</td></tr>
<tr><td>Hemiplegia</td><td>2</td><td>342-342.9 and 344.1</td></tr>
<tr><td>Moderate or severe renal disease</td><td>2</td><td>582-582.9, 583-583.7, 585, 586, and 588-588.9</td></tr>
<tr><td>Diabetes with end-organ damage</td><td>2</td><td>250.4-250.6</td></tr>
<tr><td>Any malignancy including leukemia and lymphoma</td><td>2</td><td>140-172.9,174-195.8, and 200-208.9</td></tr>
<tr><td>Moderate or severe liver disease</td><td>3</td><td>572.2-572.8 and 456.0-456.21</td></tr>
<tr><td>Metastatic solid tumor</td><td>6</td><td>196-199.1</td></tr>
<tr><td>AIDS</td><td>6</td><td>042-044.9</td></tr>
<tr><td colspan="3">c. Other definitions</td></tr>
<tr><td>Conditions</td><td colspan="2">ICD-9 codes</td></tr>
<tr><td>Major chromosomal and other congenital anomalies</td><td colspan="2">ICD-9 codes of 758.0, 758.1, 758.2, 758.3, 758.5, 758.6, 758.7, 758.8, 758.9, 759.5, 759.6, 759.7, 759.8, and 759.9</td></tr>
<tr><td>Moderate to severe intellectual disabilities</td><td colspan="2">318 and 319</td></tr>
</table>

*Adapted from Deyo et al., 1992.

Table S2. Comparison of adolescents with and without BMI measurements.

**Variable**	**Adolescents, who have measurements (N=104,887)**	**Adolescents who do not have measurements (N=154,359)**
**Sex (male),** *N (%)*	49,623 (47.3)	79,795 (51.7)
**Ethnicity (Jews),** *N (%)*	54,929 (52.4)	82,509 (53.5)
**Socio-economic status,** N (%):		

Low	38,317 (36.5)	48,529 (31.4)
Medium	48,490 (46.2)	75,774 (49.1)
High	9,501 (9.1)	18,138 (11.8)
No data	8,579 (8.2)	11,918 (7.7)
**District of residency,** N (%):		
Northern		
Haifa	17,820 (17.0)	25,842 (16.7)
Sharon-Shomron	20,961 (20.0)	29,856 (19.3)
Central	15,256 (14.5)	21,086 (13.7)
Dan-Petah-Tikva	15,014 (14.3)	21,744 (14.1)
Jerusalem	7,313 (7.0)	10,929 (7.1)
Southern	12,864 (12.3)	22,875 (14.8)
No data	15,630 (14.9)	21,980 (14.2)
	29 (0.0)	47 (0.0)

Table S3. Risk estimates of the association between the adolescent weight category and dichotomic divisions of the Charlson Comorbidity Index at age 30.

	Weight category						Total population (N=80,853)
	**Underweight (N=4,172)**	**Normal (N=57,867)**	**Overweight (N=8,883)**	**Obesity (N=7,958)**	**Class 2 Obesity (N=1,381)**	**Class 3 Obesity (N=592)**	
**a. Both sexes**							
Association of weight category in adolescence with Charlson score ≥1 at age 30							
aRR[§](95% CI)<br>p-value	0.88 (0.81–0.95)<br>0.002	Reference	1.37 (1.30–1.43)<br><0.001	1.67 (1.60–1.75)<br><0.001	2.04 (1.86–2.23)<br><0.001	2.34 (2.04–2.66)<br><0.001	
aRR[§§] (95% CI)<br>p-value	1.00 (0.92–1.09)<br>0.925		1.14 (1.08–1.19)<br><0.001	1.20 (1.14–1.27)<br><0.001	1.21 (1.09–1.34)<br><0.001	1.18 (1.02–1.36)<br>0.020	
Association of weight category in adolescence with Charlson score ≥3 at age 30							
aRR[§](95% CI)<br>p-value	0.72 (0.45–1.09)<br>0.144	Reference	1.68 (1.36–2.06)<br><0.001	2.12 (1.73–2.59)<br><0.001	4.42 (3.20–5.96)<br><0.001	4.52 (2.75–6.94)<br><0.001	
aRR[§§] (95% CI)<br>p-value	0.86 (0.53–1.32)<br>0.530		1.25 (1.01–1.55)<br>0.040	1.24 (0.99–1.55)<br>0.058	1.88 (1.31–2.64)<br><0.001	1.46 (0.85–2.36)<br>0.146	
**b. Males**							
Population	2,195	26,176	4,097	4,180	672	296	37,616
Association of weight category in adolescence with Charlson score ≥1 at age 30							
aRR[§](95% CI)<br>p-value	0.88 (0.77–1.00)<br>0.045	Reference	1.35 (1.25–1.46)<br><0.001	1.71 (1.59–1.83)<br><0.001	2.24 (1.94–2.58)<br><0.001	2.85 (2.35–3.42)<br><0.001	
aRR[§§] (95% CI)<br>p-value	1.00 (0.88–1.14)<br>0.933		1.16 (1.07–1.26)<br><0.001	1.26 (1.16–1.37)<br><0.001	1.34 (1.14–1.56)<br><0.001	1.42 (1.15–1.74)<br><0.001	
Association of weight category in adolescence with Charlson score ≥3 at age 30							
aRR[§](95% CI)<br>P-value	0.85 (0.45–1.46)<br>0.581	Reference	1.53 (1.08–2.12)<br>0.013	2.05 (1.5–2.75)<br><0.001	4.30 (2.59–6.72)<br><0.001	5.15 (2.54–9.24)<br><0.001	
aRR[§§] (95% CI)<br>p-value	0.96 (0.49–1.69)<br>0.895		1.23 (0.86–1.72)<br>0.235	1.35 (0.96–1.86)<br>0.076	2.06 (1.18–3.43)<br>0.007	1.90 (0.88–3.76)<br>0.079	
**c. Females**							
Population	1,977	31,691	4,786	3,778	709	296	43,237
Association of weight category in adolescence with Charlson score ≥1 at age 30							
aRR[§](95% CI)	0.88 (0.79–0.98)	Reference	1.38 (1.30–1.46)	1.65 (1.55–1.76)	1.91 (1.69–2.16)	1.99 (1.64–2.37)	

p-value	0.020		<0.001	<0.001	<0.001	<0.001	
aRR§§ (95% CI)<br>p-value	1.01 (0.90–1.13)<br>0.855		1.12 (1.05–1.20)<br><0.001	1.16 (1.08–1.24)<br><0.001	1.12 (0.98–1.28)<br>0.089	1.01 (0.82–1.22)<br>0.947	
Association of weight category in adolescence with Charlson score ≥3 at age 30							
aRR§(95% CI)<br>p-value	0.60 (0.28–1.09)<br>0.130	Reference	1.79 (1.36–2.32)<br><0.001	2.18 (1.65–2.85)<br><0.001	4.50 (2.91–6.65)<br><0.001	4.00 (1.90–7.32)<br><0.001	
aRR§§ (95% CI)<br>p-value	0.77 (0.36–1.41)<br>0.443		1.25 (1.95–1.65)<br>0.107	1.16 (0.85–1.57)<br>0.337	1.74 (1.08–2.72)<br>0.018	1.16 (0.53–2.27)<br>0.684	

Underweight—BMI <5th percentile, normal weight—BMI 5th-84.9th percentile, overweight—BMI 85th-94.9th percentile, obesity—BMI ≥95th percentile, not including class 2 and class 3 obesity, class 2 obesity—BMI ≥120% to <140% of the 95th percentile or BMI ≥35 to <40 kg/m^2, class 3 obesity—BMI ≥140% of the 95th percentile or BMI ≥40 kg/m^2, SD—standard deviation, IQR—interquartile range, 95% CI—95% of the confidence interval, RR—relative risk, and aRR—adjusted relative risk. §Adjusted to sex, ethnicity, socio-economic level, and district of residency. §§ Adjusted to sex, ethnicity, socio-economic level, district of residency, and adult BMI.

Table S4. Cumulative incidence of individual diseases of the Charlson Comorbidity Index at age 30, by weight category.

CCI component, N (%)	CDC weight category Underweight (N=4,172)	Normal (N=57,867)	Overweight (N=8,883)	Obesity (N=7,958)	Class 2 Obesity (N=1,381)	Class 3 Obesity (N=592)	Total population (N=80,853)
Myocardial infarction	4 (0.10)	39 (0.07)	14 (0.16)	4 (0.05)	3 (0.22)	2 (0.34)	66 (0.08)
Congestive heart failure	3 (0.07)	53 (0.09)	13 (0.15)	11 (0.14)	2 (0.14)	0 (0.00)	82 (0.10)
Peripheral vascular disease	20 (0.48)	315 (0.54)	40 (0.45)	45 (0.57)	5 (0.36)	2 (0.34)	427 (0.53)
Dementia	26 (0.62)	437 (0.76)	71 (0.80)	73 (0.92)	9 (0.65)	5 (0.84)	621 (0.77)
Cerebrovascular disease	92 (2.21)	1,530 (2.64)	272 (3.06)	262 (3.29)	68 (4.92)	27 (4.56)	2,251 (2.78)
Chronic pulmonary disease	283 (6.78)	4,531(7.83)	848 (9.55)	771 (9.69)	143 (10.35)	87 (14.70)	6,663 (8.24)
Connective tissue disease	29 (0.70)	402 (0.69)	90 (1.01)	84 (1.06)	13 (0.94)	5 (0.84)	623 (0.77)
Peptic disease	52 (1.25)	886 (1.53)	149 (1.68)	138 (1.73)	37 (2.68)	14 (2.36)	1,276 (1.58)
Mild liver disease	56 (1.34)	1,134 (1.96)	500 (5.63)	772 (9.70)	212 (15.35)	90 (15.20)	2,964 (3.67)
Diabetes without end-organ damage	28 (0.67)	770 (1.33)	252 (2.84)	397 (4.99)	118 (8.54)	54 (9.12)	1,619 (2.00)
Hemiplegia	11 (0.26)	162 (0.28)	34 (0.38)	20 (0.25)	7 (0.51)	5 (0.84)	239 (0.30)
Moderate or severe renal disease	14 (0.34)	241 (0.42)	50 (0.56)	58 (0.73)	7 (0.51)	3 (0.51)	373 (0.46)
Diabetes with end-organ damage	1 (0.02)	6 (0.01)	3 (0.03)	6 (0.08)	4 (0.29)	0 (0.00)	20 (0.02)
Any malignancy including leukemia and lymphoma	39 (0.93)	497 (0.86)	86 (0.97)	79 (0.99)	5 (0.36)	7 (1.18)	713 (0.88)
Moderate or severe liver disease	0 (0.00)	0 (0.00)	0 (0.00)	0 (0.00)	0 (0.00)	0 (0.00)	0 (0.00)
Metastatic solid tumor	0 (0.00)	0 (0.00)	0 (0.00)	0 (0.00)	0 (0.00)	0 (0.00)	0 (0.00)
AIDS	2 (0.05)	34 (0.06)	5 (0.06)	4 (0.05)	2 (0.14)	0 (0.0)	47 (0.06)

Underweight—BMI <5th percentile, normal weight—BMI 5th-84.9th percentile, overweight—BMI 85th-94.9th percentile, obesity—BMI ≥95th percentile, not including class 2 and class 3 obesity, class 2 obesity—BMI ≥120% to <140% of the 95th percentile or BMI ≥35 to <40 kg/m^2, and class 3 obesity—BMI ≥140% of the 95th percentile or BMI ≥40 kg/m^2.
